# Inherent instability leads to high costs of hovering in near-neutrally buoyant fishes

**DOI:** 10.1073/pnas.2420015122

**Published:** 2025-07-07

**Authors:** Valentina Di Santo, Xuewei Qi, Fidji Berio, Angela Albi, Otar Akanyeti

**Affiliations:** ^a^Scripps Institution of Oceanography, University of California, San Diego, La Jolla, CA 92093; ^b^Department of Zoology, Stockholm University, Stockholm 11418, Sweden; ^c^Department of Collective Behaviour, Max Planck Institute of Animal Behavior, University of Konstanz, Radolfzell am Bodensee 78315, Germany; ^d^Centre for the Advanced Study of Collective Behaviour, University of Konstanz, Konstanz 78464, Germany; ^e^Department of Biology, University of Konstanz, Konstanz 78547, Germany; ^f^Department of Computer Science, Aberystwyth University, Aberystwyth, Ceredigion SY23 3FL, Wales, United Kingdom

**Keywords:** dynamic stability, fish locomotion, hovering, inherent instability

## Abstract

Hovering, the ability to maintain a stationary position in fluid, is essential for many fish species during prey capture, habitat exploration, and mating. While traditionally assumed to be energetically inexpensive for fishes with a swim bladder, the metabolic costs and morphological factors influencing postural stability during hovering remain poorly understood. Hovering requires fishes to counteract small instabilities in position and orientation, often through continuous adjustments using their fins and body. To examine the energetic consequences of this active stabilization, we measured body posture, fin kinematics, and metabolic rates in 13 near-neutrally buoyant fish species during both hovering and resting. Our results show that hovering nearly doubles metabolic rates compared to resting, and species with greater separation between the center of mass and center of buoyancy and increased caudal fin activity exhibit higher energetic costs. In contrast, species with more posteriorly positioned pectoral fins and lower length-to-depth ratios show reduced hovering costs. Our findings demonstrate that, despite morphological traits that promote instability, fishes maintain posture and position through fine-scale fin control—at a significant energetic expense. This study suggests that hovering is a costly behavior that likely plays a key role in shaping the evolution of fish morphology and locomotor strategies.

## Significance

Hovering—the ability to maintain a stationary position in fluid—has traditionally been considered an energetically efficient behavior, with costs assumed to be close to resting levels. However, this study challenges that view, showing that hovering is far more metabolically expensive than previously thought. The high cost is driven by the need for constant fine motor control to counteract instability, requiring continuous fin movements for dynamic stability. Hovering is essential for behaviors like prey capture and nest tending, and understanding its energetic demands shifts the paradigm of how we view locomotion in near-neutrally buoyant fishes, offering new insights into the evolutionary pressures shaping behavior in complex environments.

Hovering—the ability to maintain a stationary position in a fluid medium—exemplifies the complex interplay between stability and maneuverability across different groups of animals, from insects and fishes to birds and mammals (1–4). Achieving this locomotor feat in air or water requires finely controlled movements that generate lift and stabilize the body relative to the surrounding environment (5, 6). This ability is particularly advantageous for accessing otherwise unattainable resources, such as nectar in flowers or small prey in crevices in aquatic habitats. Hovering demands a balance between maintaining position and executing quick, agile maneuvers in response to environmental stimuli or evading predators. Although stability and maneuverability operate on distinct principles, they are inherently connected: Stability corrects unintended displacements, while maneuverability enables deliberate changes in direction(7). Inherent (or static) instability is therefore crucial for achieving effective high maneuverability underwater (7).

Most fishes possess a swim bladder, making them nearly neutrally buoyant and allowing rapid adjustments to buoyancy in response to changes in depth and pressure by modulating the gas within the bladder (8). These fishes experience instability due to destabilizing forces generated by ventilation and morphological factors, such as the physical separation between their center of mass (COM) and center of buoyancy (COB). The COM is determined by the distribution of major muscle groups and the axial skeleton, typically positioned dorsally, while the COB is influenced by the location of the viscera and swim bladder, often situated ventrally. After small perturbations, whether caused by internal processes like ventilation or external forces from the environment, the COM-COB separation induces torque around the center of mass, making the fish roll and pitch involuntarily (9). Rotations result in changes in body posture that can be controlled by modulating the movement of body and fins (1, 10). Dynamically stable fishes are able to take corrective actions using their body and fins to maintain fixed position and orientation. Traditionally, hovering has been considered energetically inexpensive because the near-neutral buoyancy of many fishes reduces the need to generate lift (11). As a result, it was often assumed that at a speed = 0, metabolic rates would be comparable to resting metabolic rates in a diversity of fishes (11–13). However, this perspective may overlook the role of inherent instability, which necessitates continuous fin movements for stabilization, potentially increasing energy expenditure from resting (14). As a result, the energetic costs of maintaining a stationary position in the water column may be higher than previously thought (10, 11, 13). While stationary flight control and energetics have been well studied in aerial animals (3, 15–17), hovering in the aquatic environment has received limited attention (10, 18). Hovering is essential for prey capture, exploration, and mating in fishes, requiring continuous postural adjustments. These demands have likely shaped the evolution of fish morphology and locomotion. Understanding these dynamics reveals how fishes optimize posture, stability, and energy efficiency in complex aquatic environments (5, 19).

Here, we present a comparative study that quantified the relationship between morphology, kinematics, and energetics in a diversity of near-neutrally buoyant fish species. To investigate potential variation in hovering mechanisms and energetic consequences, we selected 13 fish species with different morphological characteristics. We hypothesized that morphological features that destabilize posture—such as a large COM-COB distance, fin position away from the COM, and a laterally compressed body shape—could drive a diversity of versatile fin movements or, alternatively, converge toward a narrower range of effective motions to achieve postural equilibrium during hovering. Similar patterns have been observed in steady swimming (20, 21) and maneuvering (7). We further expected that the inherent instability and the fin movements needed to correct postural imbalances could lead to higher energetic costs than previously anticipated (10).

## Results

**Fig. 1.**
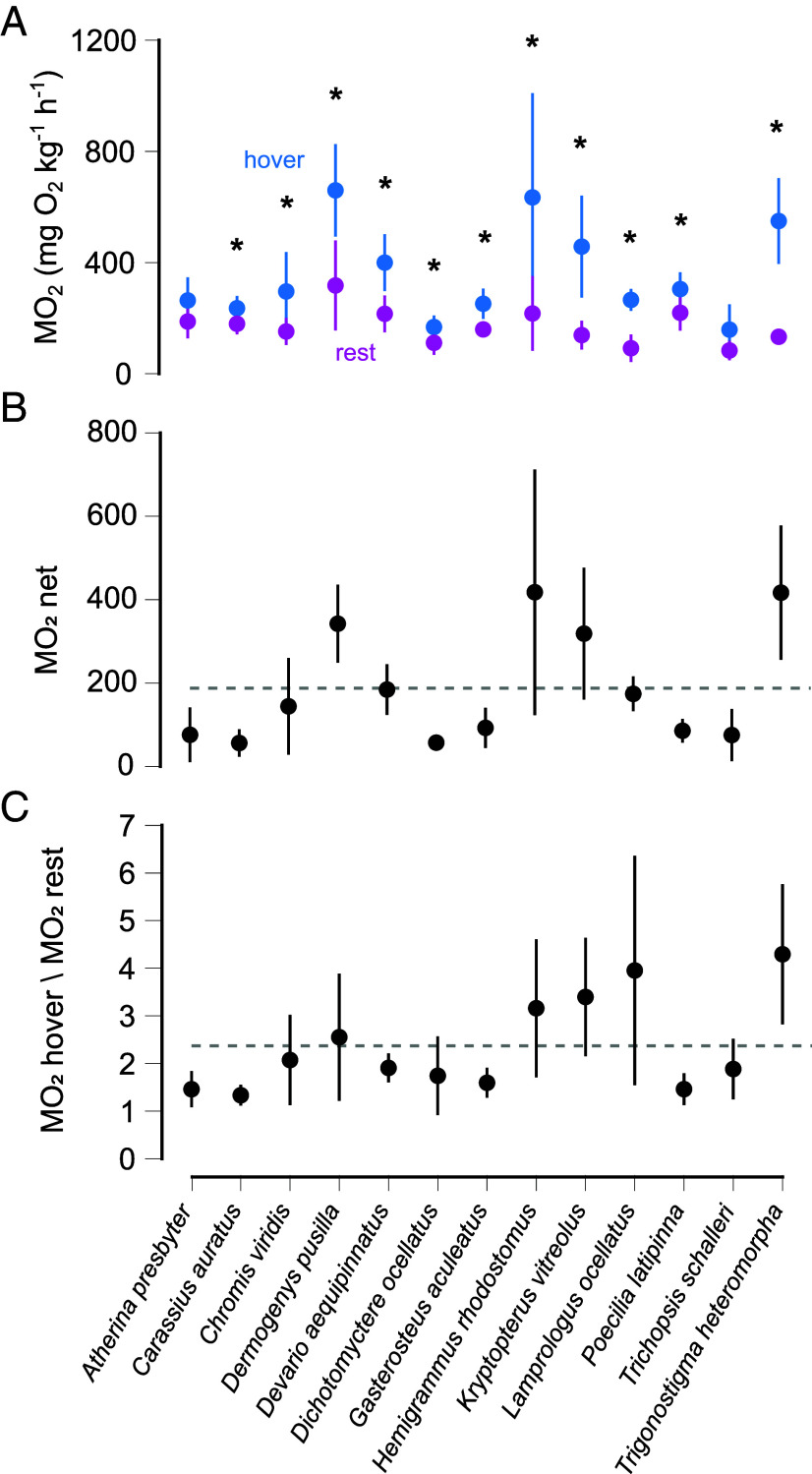
Hovering increases metabolic rates (MO_2_) from resting across species (n = 5 to 8). (*A*) MO_2hover_ was significantly elevated from MO_2rest_ for all the species except for sand smelt and three-stripe gourami (asterisks denote statistically a significant difference between MO_2hover_ and MO_2rest_ for each species, *P* < 0.05). (*B*) Species are divided into two groups based on whether their MO_2net_ is above or below the average (horizontal dashed line). (*C*) The same grouping is applied for MO_2hover_/MO_2rest_.

Hovering resulted in significantly higher metabolic rates (MO_2hover_) compared to rest (MO_2rest_) for most species, with a doubling of MO_2_ (Fig. 1 and *SI Appendix*). MO_2hover_ values ranged from 158.48 to 351.37 mgO_2_ kg^−1^ h^−1^, with significant variation across species (SI). Energetic costs ranged from 0.12 to 0.94 KJ kg^−1^ for 10 min of hovering. Species were grouped into low and high MO_2_ groups (G1 and G2, respectively) based on whether the hovering metabolic costs were below or above the average for all the species tested (Fig. 1 and *SI Appendix*, Table S1).

**Fig. 2.**
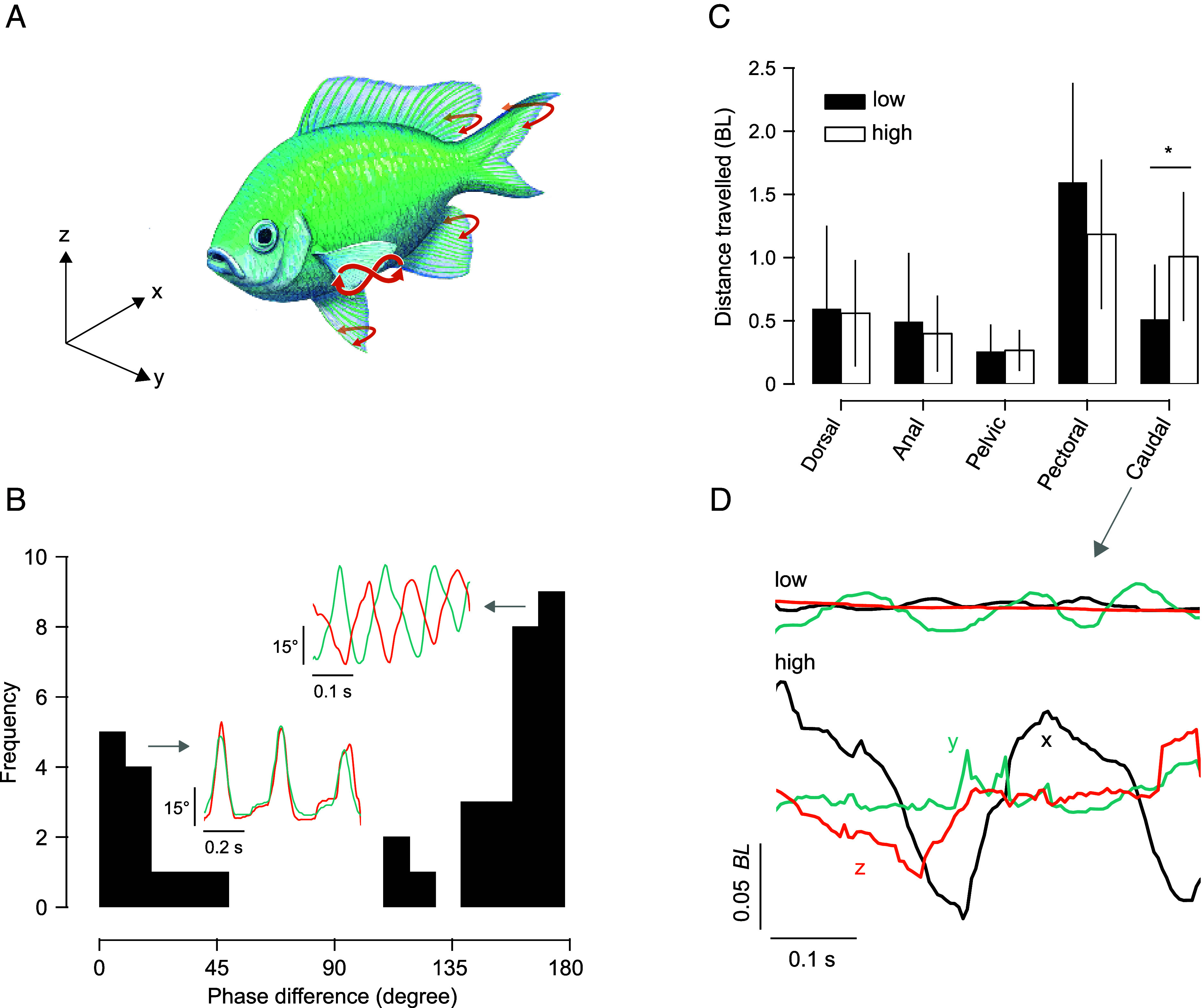
Hovering requires the movement of all fins. (*A*) 3D fin movements during hovering. (*B*) Pectoral fin movements varied between in-phase and antiphase. (*C*) The distance traveled by each fin varied across species, with significant differences in caudal fin movement between the metabolic rate group, G1, or low metabolic group (species: *Atherina presbyter*, *Carassius auratus*, *Chromis viridis*, *Dichotomyctere ocellatus*, *Gasterosteus aculeatus*, *Poecilia latipinna*, and *Trichopsis schalleri*) and G2, or high metabolic group (species: *Dermogenys pusilla*, *Devario aequipinnatus*, *Hemigrammus rhodostomus*, *Kryptopterus vitreolus*, *Lamprologus ocellatus*, and *Trigonostigma heteromorpha*). Asterisks denote a statistically significant difference between G1 and G2 (*P* < 0.05). (*D*) Example of 3D caudal fin movement in a fish from the G1 and G2 groups.

**Fig. 3.**
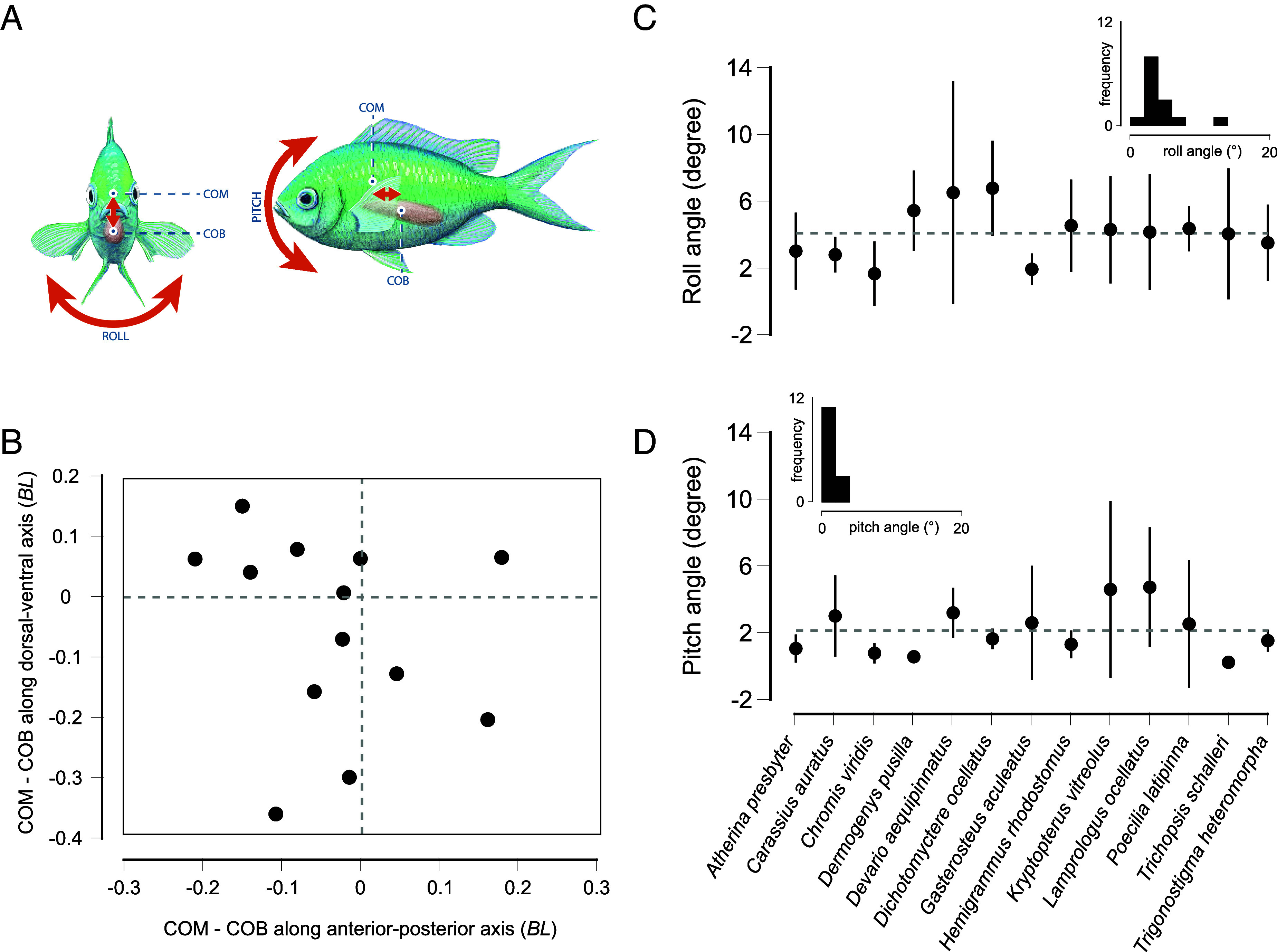
Inherent instability vs. dynamic stability during hovering. (*A*) Near-neutrally buoyant fishes are inherently unstable due to the separation of the center of mass (COM) and the center of buoyancy (COB). (*B*) Measurements show that 12 out of 13 fishes are inherently unstable on either the dorsal–ventral, anterior–posterior axes, or both, given that the COM and COB are separated. (*C*) The roll angle across species is maintained low, and most fishes have an average roll angle at or below an average of about 4° (horizontal dashed line). (*D*) The pitch angle is also low across species, and most fishes show a pitch angle at or below the average of about 2° (horizontal dashed line).

Fishes exhibited complex 3D fin movements during hovering, with caudal fin distance traveled being significantly higher in species with elevated metabolic costs (Fig. 2). The movement patterns of pectoral fins varied between in-phase and antiphase, with no significant difference in pectoral fin displacement over time across species (Fig. 2). Hovering fishes maintained stable roll and pitch angles, despite inherent instability due to the separation of their centers of mass and buoyancy (COM and COB) (Fig. 3 and *SI Appendix*, Table S1). Body angle differed across species, ranging from nearly horizontal to steep positive or negative angles. Significant variation was observed in body mass, total length, and fineness ratios (total length to maximum body depth and total length to maximum body width) across species (*SI Appendix*, Table S1).

**Fig. 4.**
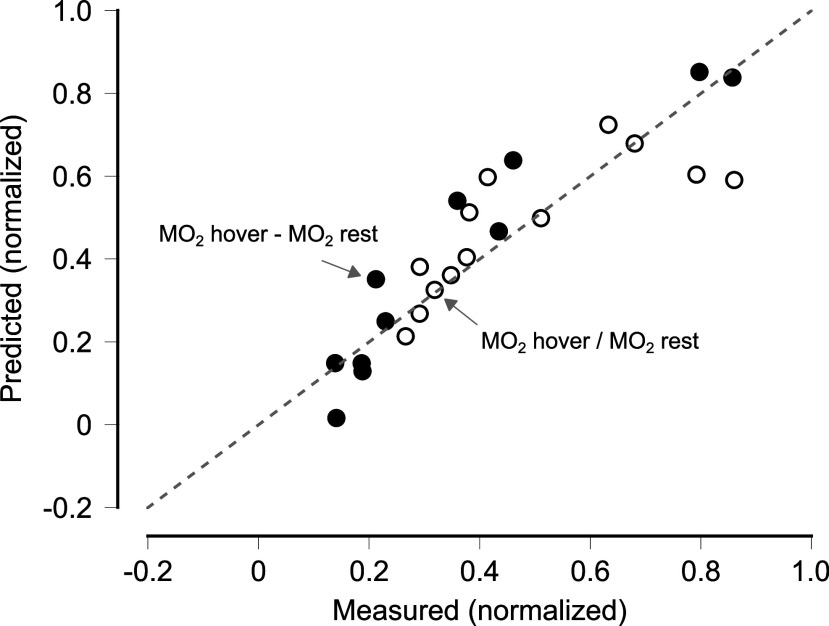
Multilinear regression model showing measured and predicted values for MO_2net_ and MO_2hover_/MO_2rest_. The model explains 86% of the variation in MO_2net_ and 63% of the variation in MO_2hover_/MO_2rest_.

The multilinear regression model revealed that body mass, fineness ratios, COM-COB, and the position of the pectoral and caudal fins significantly predicted metabolic rates during hovering, explaining 86% of the variation in MO_2net_ and 63% of the variation in MO_2hover_/MO_2rest_ (Table 1 and Fig. 4). Specifically, larger body mass was associated with lower MO_2net_ and MO_2hover_/MO_2rest_, while higher fineness ratios correlated with increased metabolic costs. A greater COM-COB separation was correlated with higher energetic costs, particularly along the dorsal–ventral axis. The reason for the regression model returning a negative coefficient for COM-COB separation was due to the selection of the most dorsal point as the origin, i.e., the distance between COM and COB is a negative number when COB is positioned more ventrally (i.e., lower) than COM. Hence, the negative coefficient represents a positive relationship between COM-COB separation and MO_2_. Fishes with more posterior pectoral fins (i.e., farther from the center of mass) exhibited lower metabolic costs of hovering.

**Table 1.** Multilinear regression coefficients for MO_2net_ and MO_2hover_/MO_2rest_

Coefficient variable (X)	MO_2_ net (Y)	MO_2hover_/MO_2rest_ (Y)
C_0_ = intercept	1,501.05	9.35
C_1_ = mass	−440.24	−2.91
C_2_ = fineness ratio (TL/MBD)	201.28	0.63
C_3_ = COM-COB (A-P)	−114.18	−0.34
C_4_ = COM-COB (D-V)	−116.80	−0.80
C_5_ = fineness ratio (TL/MBW)	194.16	0.21
C_6_ = pectoral fin position	−1,297.36	−3.94
C_7_ = caudal fin position	−1,297.60	−6.97
*R* ^2^	0.86	0.63

TL = Total Length, TL:MBD = Total Length to Maximum Body Depth ratio, COM-COB (A-P) = distance between the center of mass and buoyancy on the anterior–posterior axis as a proportion of body length, COM-COB (D-V) = distance between the center of mass and buoyancy on the dorsal–ventral axis as a proportion of body depth, TL:MBW = Total Length to Maximum Body Width ratio, pectoral and caudal fin position on the anterior–posterior axis.

Modeling the energetic demands of hovering in fishes is inherently complex, requiring the integration of body morphology, hydrodynamic forces, and three-dimensional fin kinematics. Here, we attempt to provide a simple mechanistic model linking morphology to energetic cost, thereby justifying the inclusion of specific morphological variables in our regression analysis.

The stability equation can be expressed as

[1] $$\tau_{\text{fin}}=\tau_{\text{COM-COB}}-I\alpha -D,$$

where *τ*_COM-COB_ is the torque generated by the spatial separation between COM and COB. During hovering, this is the torque that induces instability and scales linearly with body mass and distance between COM and COB (*SI Appendix*, Fig. S1). *τ*_fin_ is the torque generated by the corrective fin movements opposing *τ*_COM-COB_ to maintain posture and depends on both fin position and kinematics. All else being equal, the pectoral fins positioned farther from the center of mass have a longer lever arm, hence generating greater torque, than those positioned closer to the center of mass. To put it in another way, more distal fins will have greater leverage and need to produce smaller forces to oppose the destabilizing *τ*_COM-COB_, thus improving stabilization efficiency. However, we recognize that too much leverage can itself be destabilizing if fins overproduce these forces.

*I* (moment of inertia), which is the resistance of the body to angular acceleration (*α*), is a function of body mass (linear scaling) and radius of gyration (exponential scaling). The latter changes depending on the axis of rotation; i.e., it is proportional to body width during roll and body length during pitch. Finally, *D* is the hydrodynamic drag which opposes *τ*_COM-COB_ and depends on the surface area and angular velocity.

This equation suggests that fishes with smaller surface area (lower drag), greater COM-COB separation (greater *τ*_COM-COB_), and pectoral fins located near the center of mass (less leverage) experience increased energetic demands for stabilization. While mass affects both *τ*_COM-COB_ and *I* linearly, their counterbalancing effects suggest that mass alone does not directly dictate instability. However, assuming isometry, body size affects *I* more than *τ*_COM-COB_ (due to exponential term), making bigger fish more stable. In addition, it is likely that lighter fishes are less stable, incurring higher energetic costs due to overcorrection of the fins or oversensitivity to internal (e.g., ventilation) and external perturbations (e.g., turbulence). Hence, we predict negative regression coefficients for COM-COB (at least on the dorsal–ventral axis), fin positions, and mass, and positive coefficients for fineness ratios, as deeper and wider-bodied fish have larger surface areas (greater drag). Nevertheless, nonlinear interactions and additional factors may modulate these relationships, necessitating empirical validation.

**Fig. 5.**
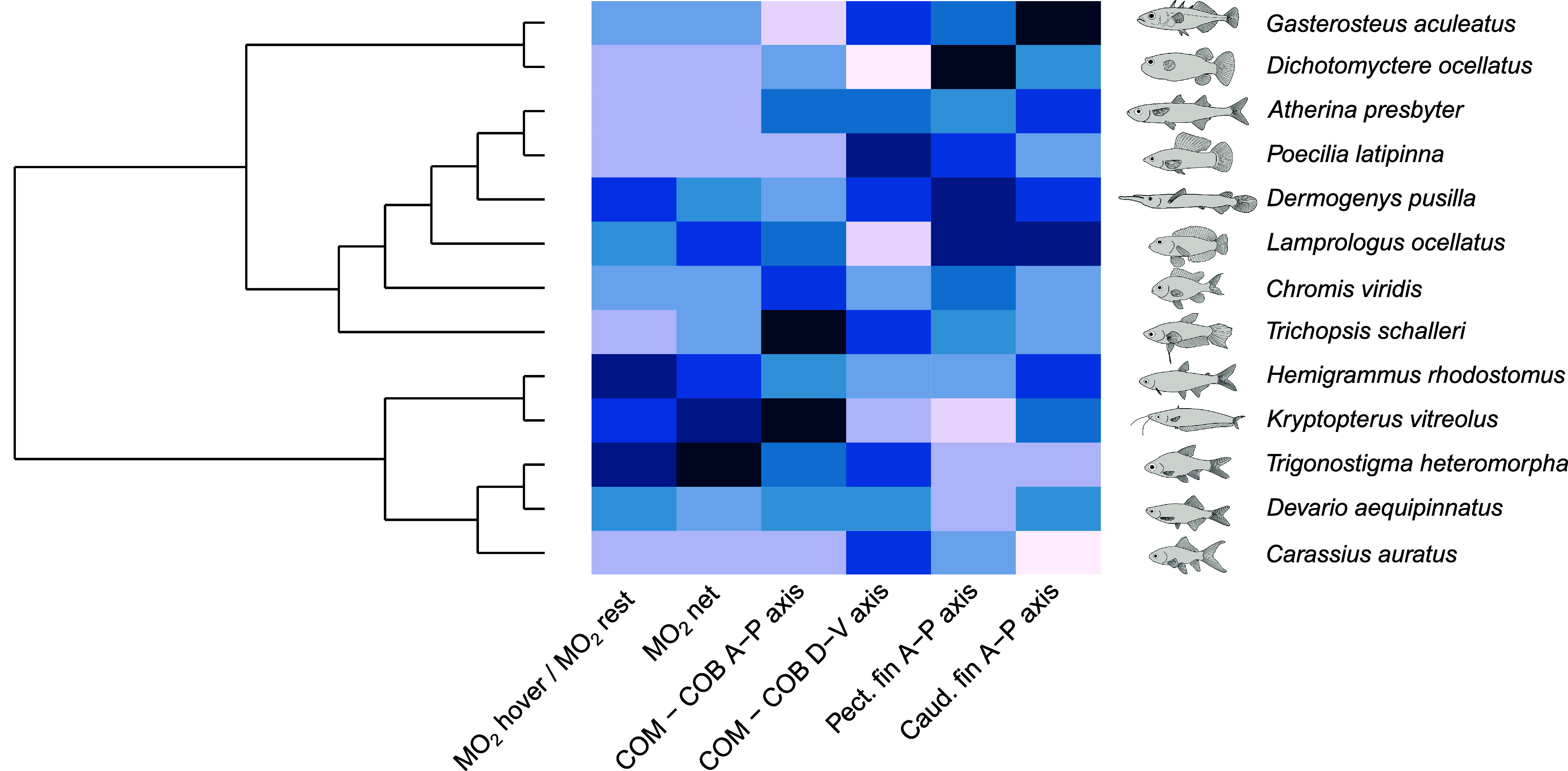
Energetic, morphological, and kinematic measurements mapped on phylogenetic relationships of fishes. MO_2hover_ / MO_2rest_, MO_2net_ = MO_2hover_ − MO_2rest_ in mgO_2_ kg^−1^ h^−1^. COM–COM A-P Axis = difference in position between the center of mass and center of buoyancy as a proportion of body length along the anterior–posterior axis. COM–COB D-V Axis = difference in position between the center of mass and center of buoyancy as a proportion of body depth along the dorsal–ventral axis. Pect. fin A-P axis = pectoral fin position along the anterior–posterior axis as a proportion of body length. Caud. fin A-P axis = caudal fin position along the anterior–posterior axis as a proportion of body length. All values have been centered and scaled for each variable. Color gradient ranges from light (low values) to dark (high values) blue. Phylogenetic relationships follow Betancur-R et al. (22).

Among the variables tested, only pectoral fin position along the anterior–posterior axis was phylogenetically structured (Fig. 5 and *SI Appendix*, Table S2). When the pectoral fin position was removed from the multiregression analysis, the model still had a high prediction performance (*R*^2^ > 0.6). This suggests that the link between morphology and energetics cost of hovering cannot be solely explained by the phylogenetic relationships. Among all the variables included in the model, only the two fineness ratios were not independent (*P* < 0.01), suggesting that in our dataset deep-bodied fishes were also wide (*SI Appendix*, Table S3).

We next evaluated the importance of each variable predicting MO_2net_ and MO_2hover_/MO_2rest_ using two different methods (by removing it from the regression analysis or randomly shuffling its values) and observing the resulting degradation in the model’s performance, which was measured by the change in *R*^2^ value (*SI Appendix*, Table S4). The results from both methods were in close agreement, indicating that mass, COM-COB separation (on both axes), pectoral fin position, and fineness ratios were the most influential morphological predictors. The results also showed that having only one fineness ratio (either TL/MBD or TL/MDW) was sufficient to maintain high performance. This was rather expected, as the two fineness ratios were highly correlated.

We ran a multilinear regression analysis, using a reduced set of predictor variables, demonstrating that the model retains strong predictive performance, with $R^{2}=0.80$ for MO_2net_ and $R^{2}=0.59$ for MO_2hover_/MO_2rest_ (*SI Appendix*, Table S5). The regression coefficients indicate that mass is a dominant negative predictor for both MO_2net_ and MO_2hover_/MO_2rest_, with coefficients of −307.43 and −2.21, respectively. Similarly, COM-COB displacements in both the anterior–posterior and dorsal–ventral directions negatively influence metabolic rates. To assess whether the position of the COB along the anterior–posterior axis relative to the COM (i.e., anterior or posterior) was important in predicting hovering metabolic rates, we ran the model using the absolute value of the anterior–posterior COM-COB displacement and found no significant change in the model *R*^2^ values (0.75 for MO_2net_, 0.65 for MO_2hover_ MO_2rest_). In contrast, the fineness ratio (TL/MBW) exhibits a positive effect, suggesting that elongated body shapes are associated with increased metabolic rates. Pectoral fin position exerts the most substantial negative effect on both response variables, with coefficients of −1,438.78 and −4.78. These findings highlight the robustness of the regression model in capturing key morphological influences on metabolic rates with high explanatory power grounded by the theory.

## Discussion

Many near-neutrally buoyant fishes are typically highly maneuverable and capable of maintaining dynamic postural equilibrium during swimming. Being inherently unstable, they use fins to generate stabilizing forces and correct rotational movements. This comparative study demonstrates that hovering is expensive in a diverse group of fishes with different morphological and ecological adaptations (Fig. 5). On average, fishes double their MO_2_ from resting to hovering. Pectoral fins can switch between in-phase and antiphase motion, yet remain synchronized for the majority of the fin beat cycles, indicating a convergence toward a small range of effective motions. We identified two groups of fishes based on MO_2hover_: a low-rate group (G1) and a high-rate group (G2). G1 fishes are characterized by larger masses or deep bodies, while G2 fishes are small, thin, and have elongated bodies, exhibiting greater caudal fin movement, and pectoral fins closer to the anterior of their body, typical of maneuvering fishes (*SI Appendix*, Table S1). Additionally, a high COM-COB distance along the anterior–posterior and dorsal–ventral axes increases MO_2hover_ (Table 1).

Much research on hovering has focused on insects, birds, and bats (4, 6, 17, 23–25), but studies on fishes are scarce and mostly descriptive (1, 26, 27). From a fluid dynamics perspective, negatively buoyant fish, birds, bats, and insects have similarities since they all need to create lift during hovering (28, 29). However, the density (*ρ*) of air is much lower than that of water ($\rho_{\text{air}}=1.3\;\text{kg}\;{\text{m}}^{-3}$, $\rho_{\text{water}}=1,000\;\text{kg}\;{\text{m}}^{-3}$), meaning that animals in water experience a much greater buoyant force. As a result, even negatively buoyant fishes have their weight partially offset by the water they displace (i.e., buoyant force = volume displaced $\times \rho_{\text{water}}\times g$). Thus, the net force that must be actively countered to hover in water is considerably smaller than the full body weight (mass × *g*), as is the case for aerial hoverers. For instance, induced power during hovering was $0.075\;\;\text{W}\;{\text{N}}^{-1}$ in mandarin fish (Synchropus picturatus), a negatively buoyant fish (28), and was relatively low compared to bumble bee (*Bombus terrestris*) ($2.1\;\;\text{W}\;{\text{N}}^{-1}$ (4)), and pigeon (*Columba livia*) ($2.2\;\;\text{W}\;{\text{N}}^{-1}$ (23)). In fishes, the energetic cost of hovering therefore comprises both lift generation (where needed) and the continuous fine motor control required to maintain postural stability (10, 30). By focusing on near-neutrally buoyant species, our study isolates the cost of postural control and demonstrates that it is a significant contributor to elevated metabolic rates during hovering.

Hovering is a widespread behavior observed across many fish species (5, 31), and is often associated with station-holding near physical structures such as corals or the benthic substrate (27, 32). When hovering occurs within approximately 1 cm of a surface, fishes can reduce the power required for station-holding by 30 to 60% (28), likely due to hydrodynamic interactions with the boundary layer. In the present study, the high costs of hovering did not originate from the need to create hydrodynamic lift but rather from the need to counteract morphological instability and perhaps internal perturbations (i.e., ventilation). It is the impressive dynamic stability that fishes exhibit, despite their inherent instability, that necessitates multiple propulsors to move continuously to remain “in place,” resulting in high metabolic costs.

Previous studies have described the general motions of near-neutrally buoyant fishes while hovering. Drucker and Lauder (33) described the movement of the pectoral fins in rainbow trout (*Oncorhynchus mykiss*) as depressed beneath the body and twisted while sculling. Rainbow trout move their pectoral fins synchronously but antiphase (33). Pectoral fins create a rotational moment around the COM during each half of the 8-shaped cycle, which is balanced across the full fin cycle, reducing the yawing of the body during hovering (33). In a study on bluegill sunfish (*Lepomis macrochirus*), Kahn et al. (26) reported that these fish exhibit a variety of kinematic patterns during hovering, not easily quantifiable using the same metrics as those for translational locomotion (20, 34, 35). Sunfish bluegill use their pectoral fins in symmetric or asymmetric cupping motion, or as a flat plate during the instroke motion (26, 36). In the present study, fishes used their pectoral fins continuously to create complex 3D motion, including 8-shaped trajectories. We found that the pectoral fins traveled a distance of up to about 2.5 BL in one second, while the other fins moved much less (1 BL or less in one sec, Fig. 2). While it is apparent that the pectoral fins move continuously and in a complex pattern to allow the fish to stay “in place,” it is the caudal fin movement that differs between the high and low MO_2_ groups. The caudal fin is key during steady and unsteady swimming in the majority of fishes (9, 37, 38) and might stabilize the body during hovering as well (36, 39). During unsteady maneuvers, such as hovering and forward-sinking, parrot cichlid (*Cichlasoma* sp.) execute caudal fin-wave propagation (40). The caudal fin undergoes significant deformation to allow a wave of bending to pass across the fin surface, thereby facilitating pitching stabilization of the fish (40). In our study, fishes with the greatest inherent instability exhibited increased caudal fin movement, leading to higher MO_2hover_. The caudal fin, being large and costly to actuate, contributes to high locomotion costs (41), as confirmed by our data.

The caudal fin is not the only propulsor used for body stabilization; pelvic, anal, and dorsal fins also contribute (1, 36) (Fig. 2). Pelvic fins are used to actively control posture at low velocities by oscillating and slowing the flow along the ventral body, affecting pitch and yaw perturbations (42). This movement generates trim correction forces during unstable maneuvers (43). Additionally, pelvic fins influence the anal fin’s angle of attack and help damp body oscillation, providing drag for stabilization during slow-speed swimming (42). The anal and dorsal fins, positioned above and below the COM, produce balancing torques that stabilize the body (34, 44). For instance, the ribbonfin, used by knifefishes for propulsion and maneuvering, extends along the body and consists of numerous fin rays that undulate in a wave-like motion. This undulatory motion allows precise control and maneuverability, enabling the fish to move forward, backward, and hover in place. Hovering in ghost knifefish (*Apteronotus albifrons*) is achieved by generating two counterpropagative waves, one from the caudal edge and the other from the rostral edge (1). These waves meet at a nodal point near the middle of the fin, canceling translational movement and enhancing stabilization (1).

Inherent instability, while energetically costly, represents a biomechanical trade-off enabling enhanced maneuverability, an advantageous trait in structurally complex aquatic habitats. The evolution of the swim bladder facilitated diversified fin positioning, thereby supporting the extensive radiation of locomotor morphologies in teleost fishes (45). Instability thus provides a functional reference upon which control systems can effectively operate (5, 36, 46). Our findings challenge the widespread view that hovering is energetically inexpensive (11, 14). Instead, we demonstrate that the need to counteract the inherent instability caused by the separation of COM and COB requires continuous fine motor control, leading to higher energy expenditure. This shift in our understanding of the energetic demands of hovering reveals that dynamic stability, while essential, comes at a significant metabolic cost. These findings suggest that hovering may play a more influential role in shaping fish morphology and biomechanics than previously thought, with important implications for how fish balance the trade-offs between stability and energetic efficiency. The survival and growth of fishes depend on their ability to swiftly and effectively respond to internal and external stimuli (31). For instance, fishes may quickly change direction to avoid predators or follow conspecifics in a school (47). Although maneuvers are energetically expensive, they are essential and widespread behaviors. Near-neutrally buoyant fishes, with their bodies mostly supported by water, can use their fins as control surfaces (5). In this study, we found that the position of the pectoral fins affected the energetic costs of hovering. In particular, we found that fishes with more posterior pectoral fins had lower MO_2net_ as pectoral fins act as stabilizers (48, 49). Fishes are constantly engaging these control surfaces to create counteracting and stabilizing forces, in addition to generating thrust (37).

While maneuvering has been well studied in fishes, stabilization motions are less apparent. Stability limits maneuverability, yet studies have shown that most fishes are both highly maneuverable and dynamically stable (50). Our study shows that both roll and pitch angles in near-neutrally buoyant fishes while hovering are comparable to those during steady swimming (<10^°^) (51). Webb (7) suggested that postural adjustments when fishes are not resting on a substrate may constitute up to 10% of the total energy costs of swimming. Our study analyzes the costs of stabilization during hovering in a 13 fish species. We found that, in the investigated species, stabilization costs range from 0.1 to 3 times those of resting, which is in agreement with some of the costs hypothesized by Webb. Unsurprisingly, hovering specialists such as gourami, stickleback, and pufferfish have low energetic costs of hovering, while giant danio, cichlid, and glass catfish have exceptionally high metabolic rates during hovering. While giant danio spend much of their time moving and rarely swim in place, cichlids and glass catfish often hover. Frog-faced cichlid need to hover over their nests to protect their offspring (52). Perhaps cichlids use the interaction with the substrate to reduce energy expenditure during hovering, as seen in mandarin fish (28). In our study, fishes hovered off the substrate and away from the walls to reduce ground and wall effects (32), so we might have restricted the employment of clever behavioral adjustments that save energy through body–substrate interactions. However, this elevation from the substrate provides a more objective estimation of hovering costs. While glass catfish hover in school formations, they might harvest vortices shed by conspecifics to save energy (18, 53). Further research is necessary to elucidate the hydrodynamic consequences of instability during hovering. Our results indicate that hovering is a costly maneuver, for instance requiring, on average, 464% more energy than swimming at 0.5 BL s^−1^ in eel (*Anguilla anguilla*) and 45% more than swimming at 0.25 BL s^−1^ in rainbow trout (10, 54). These high costs are the consequence of the need to balance inherent instability with precise control of body posture.

## Conclusions

This comparative study elucidates the mechanics and energetic costs associated with hovering in near-neutrally buoyant fishes. Our results show that hovering elevates metabolic rates significantly, with substantial interspecific variation influenced by morphological characteristics and fin movements. Smaller fishes with greater caudal fin activity and higher fineness ratios experience higher energetic costs during hovering. Despite their inherent instability, caused by morphological characteristics such as the spatial separation of COM and COB, near-neutrally buoyant fishes achieve dynamic stability through continuous fin adjustments. Destabilizing forces are mitigated by hydrodynamic friction and inertia of the fish’s morphology. For instance, deep-bodied fish, with a larger surface area, experience increased hydrodynamic friction, reducing the impact of destabilizing forces, while greater mass increases inertia, further dampening these effects.

This study shows that hovering can be energetically expensive, revealing that the need to counteract instability comes with considerable metabolic costs. This underscores the complex interplay between instability and the use of control surfaces to maintain position. This study also advances our understanding of the biomechanical and energetic constraints of hovering, offering key insights into the adaptations that allow fishes to remain stationary while highlighting the inherent trade-offs between maneuverability and energetic efficiency. Future research may explore the ecological implications of these energetic demands and investigate strategies fishes may employ to optimize energy efficiency during hovering and maneuvering.

## Materials and Methods

### Animal Husbandry.

Individuals from each of the 13 species of fish (n = 4 to 8 for metabolic measurements and n = 3 for kinematic analyses) (*SI Appendix*, Table S1) were obtained from vendors (Imazo, Sweden, DeJong Marine, The Netherlands, and Flying Sharks, Portugal) or wild-caught, and imported to Sweden with permit no. 6.6.18-11773/2019. All fishes were maintained at the Department of Zoology at Stockholm University. All husbandry and experimental procedures followed the Animal Ethics Protocol (no. 11924-2020) approved by the Swedish Board of Agriculture. The fishes were fed a diet of frozen fish, squid, shrimp, or flakes ad libitum, but were fasted for at least 24 h before experimentation to ensure postabsorptive state for all metabolic measurements during hovering and resting.

### Energetic Measurements.

Dissolved oxygen was measured using an optical oxygen meter (FireSting-O2, PyroScience, Germany) calibrated with 100% air-saturated water. Fishes were individually transferred to a 0.615 L glass respirometer chamber and allowed to acclimate for 2 h before trials, with the chamber open and water continuously aerated. As the species used in this study are social, the respirometers were positioned adjacent to each other, and dim lighting was provided to allow the fish visual contact with their conspecifics, thereby reducing stress. Following acclimation, fishes remained in the chamber for 1 h while oxygen consumption was recorded every minute to determine metabolic rates during hovering (MO_2hover_). All fishes hovered for at least 30 min. MO_2hover_ was calculated from the slope of oxygen decline over time in the respirometer using the formula:

$${\text{MO}}_{2}=\frac{\Delta [\;{\text{O}}_{2}]}{\Delta t}\times V\times M^{-0.9},$$

where $\Delta [{\text{O}}_{2}]$ is the change in oxygen concentration (mg O_2_ L^-1^), Δt is the change in time (min), *V* is the volume of water in the respirometer chamber (L) minus the fish volume, *M* is the fish mass (kg), and 0.9 is the scaling coefficient correcting for the allometric relationship between metabolic rates and mass (55, 56). To quantify resting metabolic rates (MO_2rest_), individual quiescent fishes were placed in the respirometer chamber for about 1 h. As most fishes hovered continuously, a small amount of MS-222 (0.02 g L^-1^) was added to the water to lightly sedate the fishes, ensuring they rested and exhibited no fin movement without being fully anesthetized. Using low-dose MS-222 to calculate resting metabolic rates has been validated in previous work for fishes that do not cease moving in the respirometer chamber (57). These studies show that MS-222 does not significantly affect metabolic rates in fishes (57–59). To quantify the net energy used to hover (MO_2net_), MO_2rest_ was subtracted from MO_2hover_ for each fish. To account for differences in size, salinity, and temperature across the species, the ratio MO_2hover_/MO_2rest_ was calculated to measure the relative energy expenditure for hovering compared to resting (56). The net energy required to hover for 10 min was then converted to KJ kg^−1^ using an oxy-calorific equivalent of 3.25 cal per 1 mg O_2_ (60). The species were classified into two groups based on their metabolic rates (MO_2net_ and MO_2hover_/MO_2rest_). Group 1 (G1), which includes species with low MO_2net_ and MO_2hover_/MO_2rest_, consists of *Atherina presbyter*, *Carassius auratus*, *Chromis viridis*, *Dichotomyctere ocellatus*, *Gasterosteus aculeatus*, *Poecilia latipinna*, and*Trichopsis schalleri*. Group 2 (G2), which includes species with high MO_2net_ or MO_2hover_/MO_2rest_, consists of *Dermogenys pusilla*, *Devario aequipinnatus*, *Hemigrammus rhodostomus*, *Kryptopterus vitreolus*, *Lamprologus ocellatus*, and *Trigonostigma heteromorpha*. If either MO_2_ net or MO_2hover_/MO_2rest_ values were in the higher category, the species was considered in the high group.

### Kinematic Data Collection.

Hovering experiments were conducted in a 52-L recirculating flow tank respirometer (Loligo Systems^®^) maintained at the appropriate temperature and salinity for each species, at flow speed = 0. To elicit hovering behavior, the fishes were individually placed in an open, optically transparent Plexiglass container within the flow tank, as described in previous work (1). The container served as a refuge for most fish species; its volume was 0.465 L, nearly 100 times larger than the largest species used in this study to avoid interactions between the fish and the walls. Ventral and lateral views were recorded using two synchronized and orthogonal high-speed cameras (1080p, Chronos Camera 2.1, Krontech) at 1,000 fps. Each video was calibrated with a 3D calibration plate using DLTdv (v.8) in MATLAB (Mathworks) (61). For each fish, a sequence of at least three complete fin cycles was extracted. Points on the body and fins of each fish were digitized and 3D coordinates of points on the body were extracted for analysis. In particular, we digitized 15 points on the fishes (tip of the snout, eye, bottom of the eye, dorsal fin tip, each pectoral and pelvic fin base and tip, anal fin tip, caudal peduncle, caudal fin tip). These points were used to quantify roll and pitch angles (°), as the max excursion on the y and z axes for two points on the eye (roll) and the snout-peduncle (pitch). Body angle (°), the angle between the fish body and the horizontal plane, was measured using the three-dimensional distance between the x, y, and z coordinates of the eye and the midpoint of the caudal peduncle. Body curvature (*κ*) was measured using the equation

[2] $$\kappa =\left|\frac{\mathrm{dT}}{\mathrm{ds}}\right|,$$

where *s* is the arc length of a curve connecting all three points in the transect and *T* is the unit tangent vector of that curve using snout, peduncle, and caudal fin coordinates. Synchronization of the right and left pectoral fins during hovering was evaluated by calculating the phase lag between the fins, which ranged from 0^°^ (in-phase, where both fins reached the maximum abducted position simultaneously) to 180^°^ (antiphase, where one fin was maximally abducted while the other was maximally adducted). To estimate the phase lag, fin movements were cross-correlated, and the time shift required to achieve maximum correlation was measured. We also calculated the distance traveled by each fin and then standardized this distance to a one-second interval.

### Morphological Measurements.

Measurements from individuals (n = 4 to 5) from each species were obtained from thawed frozen specimens or the Swedish Museum of Natural History (NRM no. 17318, 18927, 30004, 56788). From each individual, we obtained linear measurements: mass, total length (TL), maximum body depth (MBD), and maximum body width (MBW) used to calculate dimensionless numbers such as fineness ratios (TL/MBD or MBW). The COM and COB of each specimen were quantified through distinct methodological approaches to characterize their spatial distributions within the body. The COM was determined by suspending each specimen from multiple anatomical landmarks (e.g., caudal peduncle, posterior-dorsal region of the cranium, ventral midline) and capturing equilibrium positions via digital imaging (62). A vertical reference axis was overlaid on each image, extending from the suspension point, and the images were subsequently coregistered based on body morphology. The COM was identified at the intersection of the three reference axes. Its position was then expressed as a proportion of body length, calculated as the ratio of the snout-to-COM distance to the snout-to-caudal peduncle distance, and as a proportion of body depth, determined as the ratio of the dorsal surface-to-COM distance to the dorsal–ventral span at the COM. The COB was established via microcomputed tomography (μCT) of specimens preserved in 70% ethanol and chemically contrasted with Lugol’s iodine solution to enhance visualization of the swim bladder (63). Fishes were CT-scanned head-down (64). Scanning was performed using a Zeiss Xradia Versa 520 μCT system at an isotropic voxel resolution of 27.7 to 57.1 μm (100 kV, 90 mA). Image acquisition and volumetric reconstruction were executed using the Scout-and-Scan Control System and Reconstruction Software (v.16.1). The swim bladder was digitally segmented in 3D Slicer (v.5.2.2) (65), and its centroid (COB) was computed as the mean X, Y, and Z coordinates of the segmented volume using R (66). The COB was then spatially registered to the whole-body scan, and its position was quantified in the same proportional reference frame as the COM, relative to both body length and body depth (*SI Appendix*, Table S1).

### Model and Statistical Analysis.

Descriptive statistics were computed for kinematic variables and MO_2_. Mean values were compared across species using a one-way ANOVA (*α* = 0.05). Comparisons of MO_2hover_ and MO_2net_ within each species were also conducted using one-way ANOVAs (*α* = 0.05). Species were categorized into low and high MO_2_ groups based on Tukey’s HSD post hoc analysis. A *t* test was performed to compare the mean MO_2_ values between high and low groups for both MO_2net_ and MO_2hover_/MO_2rest_. Species were classified into the high group if either their MO_2net_ or MO_2hover_/MO_2rest_ values were significantly higher than the mean, with significance determined by a *P* < 0.05. All analyses were performed using Python (v.3.9.12) and JMP Pro (v.16, SAS). A multilinear regression analysis was conducted to examine the effects of morphological parameters on MO_2net_ and MO_2hover_/MO_2rest_, aiming to identify the factors contributing to increased metabolic rates. The phylogenetic structure of variables was assessed with Abouheif’s tests based on Moran’s I tests (67, 68), which do not require branch lengths and rather use tree topology. The Abouheif’s C-statistic and *P* values were calculated with the R package adephylo (v.1.1.16) (69) and phylogeny rendering was achieved with the R package phylosignal (v.1.3.1) (70).

## Supplementary Material

Appendix 01 (PDF)

## Data Availability

Data supporting this work have been deposited in Dryad. Dataset DOI: 10.5061/dryad.9cnp5hqw7 (71).

## References

[1] R. Ruiz-Torres, O. M. Curet, G. V. Lauder, M. A. MacIver, Kinematics of the ribbon fin in hovering and swimming of the electric ghost knifefish. J. Exp. Biol. **216**, 823–834 (2013). 23197089 10.1242/jeb.076471

[2] H. R. Vejdani, D. B. Boerma, S. M. Swartz, K. S. Breuer, The dynamics of hovering flight in hummingbirds, insects and bats with implications for aerial robotics. Bioinspir. Biomim. **14**, 016003 (2018). 30411710 10.1088/1748-3190/aaea56

[3] D. R. Warrick, B. W. Tobalske, D. R. Powers, Aerodynamics of the hovering hummingbird. Nature **435**, 1094–1097 (2005). 15973407 10.1038/nature03647

[4] T. Weis-Fogh, Quick estimates of flight fitness in hovering animals, including novel mechanisms for lift production. J. Exp. Biol. **59**, 169–230 (1973).

[5] P. W. Webb, D. Weihs, Stability versus maneuvering: Challenges for stability during swimming by fishes. Integr. Comp. Biol. **55**, 753–764 (2015). 26002562 10.1093/icb/icv053

[6] C. P. Ellington, M. J. Lighthill, The aerodynamics of hovering insect flight. III. Kinematics. Philos. Trans. R. Soc. Lond. B Biol. Sci. **305**, 41–78 (1997).

[7] P. W. Webb, Stability and maneuverability. Fish Physiol. **23**, 281–332 (2005).

[8] E. Strand, C. Jørgensen, G. Huse, Modelling buoyancy regulation in fishes with swimbladders: Bioenergetics and behaviour. Ecol. Model. **185**, 309–327 (2005).

[9] R. E. Shadwick, G. V. Lauder, Fish Physiology: Fish Biomechanics (Elsevier, 2006).

[10] V. Di Santo, C. P. Kenaley, G. V. Lauder, High postural costs and anaerobic metabolism during swimming support the hypothesis of a u-shaped metabolism-speed curve in fishes. Proc. Natl. Acad. Sci. U. S. A. **114**, 13048–13053 (2017). 29158392 10.1073/pnas.1715141114 PMC5724281

[11] D. Chabot, J. Steffensen, A. Farrell, The determination of standard metabolic rate in fishes. J. Fish Biol. **88**, 81–121 (2016). 26768973 10.1111/jfb.12845

[12] C. Sepulveda, J. Graham, D. Bernal, Aerobic metabolic rates of swimming juvenile mako sharks, *Isurus oxyrinchus*. Mar. Biol. **152**, 1087–1094 (2007).

[13] D. G. Roche, S. A. Binning, Y. Bosiger, J. L. Johansen, J. L. Rummer, Finding the best estimates of metabolic rates in a coral reef fish. J. Exp. Biol. **216**, 2103–2110 (2013). 23470659 10.1242/jeb.082925

[14] V. Di Santo, E. Goerig, Swimming smarter, not harder: Fishes exploit habitat heterogeneity to increase locomotor performance. J. Exp. Biol. JEB247918, 228 (2025). 10.1242/jeb.247918 PMC11993249 39973198

[15] B. Goller, D. L. Altshuler, Hummingbirds control hovering flight by stabilizing visual motion. Proc. Natl. Acad. Sci. U.S.A. **111**, 18375–18380 (2014). 25489117 10.1073/pnas.1415975111 PMC4280641

[16] P. Chai, R. Dudley, Limits to vertebrate locomotor energetics suggested by hummingbirds hovering in Heliox. Nature **377**, 722–725 (1995).

[17] T. Weis-Fogh, Energetics of hovering flight in hummingbirds and in *Drosophila*. J. Exp. Biol. **56**, 79–104 (1972).

[18] V. Di Santo, EcoPhysioMechanics: Integrating energetics and biomechanics to understand fish locomotion under climate change. Integr. Comp. Biol. **62**, 711–720 (2022). 35759407 10.1093/icb/icac095 PMC9494520

[19] V. Di Santo, Intraspecific variation in physiological performance of a benthic elasmobranch challenged by ocean acidification and warming. J. Exp. Biol. **219**, 1725–1733 (2016). 27026716 10.1242/jeb.139204

[20] V. Di Santo , Convergence of undulatory swimming kinematics across a diversity of fishes. Proc. Natl. Acad. Sci. U.S.A. **118**, e2113206118 (2021). 34853171 10.1073/pnas.2113206118 PMC8670443

[21] O. Akanyeti , Fish-inspired segment models for undulatory steady swimming. Bioinspir. Biomim. **17**, 046007 (2022). 10.1088/1748-3190/ac6bd6 35487201

[22] R. Betancur-R , Phylogenetic classification of bony fishes. BMC Evol. Biol. **17**, 1–40 (2017). 28683774 10.1186/s12862-017-0958-3 PMC5501477

[23] C. J. Pennycuick, Power requirements for horizontal flight in the pigeon Columba livia. J. Exp. Biol. **49**, 527–555 (1968).

[24] J. Håkansson, A. Hedenström, Y. Winter, L. C. Johansson, The wake of hovering flight in bats. J. R. Soc. Interface **12**, 20150357 (2015). 26179990 10.1098/rsif.2015.0357 PMC4535406

[25] U. M. Norberg, T. H. Kunz, J. F. Steffensen, Y. Winter, O. V. Helversen, The cost of hovering and forward flight in a nectar-feeding bat, *Glossophaga soricina*, estimated from aerodynamic theory. J. Exp. Biol. **182**, 207–227 (1993). 8228780 10.1242/jeb.182.1.207

[26] J. C. Kahn, B. E. Flammang, J. L. Tangorra, “Hover kinematics and distributed pressure sensing for force control of biorobotic fins” in *2012 IEEE/RSJ International Conference on Intelligent 658 Robots and Systems* (IEEE, 2012), pp. 1460–1466.

[27] J. A. Walker, M. W. Westneat, Kinematics, dynamics, and energetics of rowing and flapping propulsion in fishes. Integr. Comp. Biol. **42**, 1032–1043 (2002). 21680385 10.1093/icb/42.5.1032

[28] R. W. Blake, The energetics of hovering in the mandarin fish (*Synchropus picturatus*). J. Exp. Biol. **82**, 25–33 (1979).

[29] G. A. Bartholomew, J. R. Lighton, Oxygen consumption during hover-feeding in free-ranging anna hummingbirds. J. Exp. Biol. **123**, 191–199 (1986). 3746193 10.1242/jeb.123.1.191

[30] V. Di Santo, C. P. Kenaley, Skating by: Low energetic costs of swimming in a batoid fish. J. Exp. Biol. **219**, 1804–1807 (2016). 27080535 10.1242/jeb.136358

[31] P. W. Webb, “Maneuverability” in *Encyclopedia of Fish Physiology (Second Edition)*, S. L. Alderman, T. E. Gillis, Eds. (Academic Press, ed. 2, 2024), pp. 607–613).

[32] E. Blevins, G. V. Lauder, Swimming near the substrate: A simple robotic model of stingray locomotion. Bioinspir. Biomim. **8**, 016005 (2013). 23318215 10.1088/1748-3182/8/1/016005

[33] E. G. Drucker, G. V. Lauder, Function of pectoral fins in rainbow trout: Behavioral repertoire and hydrodynamic forces. J. Exp. Biol. **206**, 813–826 (2003). 12547936 10.1242/jeb.00139

[34] L. Wen , Understanding fish linear acceleration using an undulatory biorobotic model with soft fluidic elastomer actuated morphing median fins. Soft Robot. **5**, 375–388 (2018). 29634444 10.1089/soro.2017.0085

[35] J. Guo , Vortex dynamics and fin-fin interactions resulting in performance enhancement in fish-like propulsion. Phys. Rev. Fluids **8**, 073101 (2023).

[36] R. Williams IV, M. E. Hale, Fin ray sensation participates in the generation of normal fin movement in the hovering behavior of the bluegill sunfish (*Lepomis macrochirus*). J. Exp. Biol. **218**, 3435–3447 (2015). 26347560 10.1242/jeb.123638

[37] G. V. Lauder, E. G. Drucker, Morphology and experimental hydrodynamics of fish fin control surfaces. IEEE J. Ocean. Eng. **29**, 556–571 (2004).

[38] R. Bainbridge, Caudal fin and body movement in the propulsion of some fish. J. Exp. Biol. **40**, 23–56 (1963).

[39] J. Nursall, The caudal fin as a hydrofoil. Evolution **12**, 116–120 (1958).

[40] S. Ting, J. Yang, Pitching stabilization via caudal fin-wave propagation in a forward-sinking parrot cichlid (*Cichlasoma citrinellum* × *Cichlasoma synspilum*). J. Exp. Biol. **211**, 3147–3159 (2008). 18805814 10.1242/jeb.020263

[41] G. Iosilevskii, Locomotion of neutrally buoyant fish with flexible caudal fin. J. Theor. Biol. **399**, 159–165 (2016). 27067246 10.1016/j.jtbi.2016.04.001

[42] E. Standen, Muscle activity and hydrodynamic function of pelvic fins in trout (*Oncorhynchus mykiss*). J. Exp. Biol. **213**, 831–841 (2010). 20154199 10.1242/jeb.033084

[43] E. Standen, Pelvic fin locomotor function in fishes: Three-dimensional kinematics in rainbow trout (*Oncorhynchus mykiss*). J. Exp. Biol. **211**, 2931–2942 (2008). 18775930 10.1242/jeb.018572

[44] E. Standen, G. V. Lauder, Dorsal and anal fin function in bluegill sunfish *Lepomis macrochirus*: Three-dimensional kinematics during propulsion and maneuvering. J. Exp. Biol. **208**, 2753–2763 (2005). 16000544 10.1242/jeb.01706

[45] W. A. Gosline, “Functional morphology and classification of teleostean fishes” in *Functional Morphology and Classification of Teleostean Fishes* (University of Hawaii Press, 1971).

[46] W. W. Schultz, P. W. Webb, Power requirements of swimming: Do new methods resolve old questions? Integr. Comp. Biol. **42**, 1018–1025 (2002). 21680383 10.1093/icb/42.5.1018

[47] V. Di Santo, “Schooling in fishes” in *Encyclopedia of Fish Physiology (Second Edition)* (Academic Press, 2024), vol. 2, pp. 614–625.

[48] D. Weihs, Stability of aquatic animal locomotion. Cont. Math **141**, 443–461 (1993).

[49] L. Eidietis, T. Forrester, P. Webb, Relative abilities to correct rolling disturbances of three morphologically different fish. Can. J. Zool. **80**, 2156–2163 (2002).

[50] F. Jing, E. Kanso, Stability of underwater periodic locomotion. Regul. Chaot. Dyn. **18**, 380–393 (2013).

[51] J. E. Ciancio , Extreme roll angles in argentine sea bass: Could refuge ease posture and buoyancy control of marine coastal fishes? *Mar. Biol.* **163**, 1–11 (2016).

[52] S. Balshine, M. E. Abate, “Parental care in cichlid fishes” in *The Behavior, Ecology and Evolution of Cichlid Fishes*, M. Abate, D. Noakes, Eds. (Springer, Dordrecht, ed. 1, 2021), pp. 541–586.

[53] D. Weihs, Hydromechanics of fish schooling. Nature **241**, 290–291 (1973).

[54] V. van Ginneken , Eel migration to the sargasso: Remarkably high swimming efficiency and low energy costs. J. Exp. Biol. **208**, 1329–1335 (2005). 15781893 10.1242/jeb.01524

[55] C. L. Jerde , Strong evidence for an intraspecific metabolic scaling coefficient near 0.89 in fish. Front. Physiol. **10**, 1166 (2019). 31616308 10.3389/fphys.2019.01166 PMC6763608

[56] A. Clarke, N. M. Johnston, Scaling of metabolic rate with body mass and temperature in teleost fish. J. Anim. Ecol. **68**, 893–905 (1999).

[57] V. Di Santo, Ocean acidification exacerbates the impacts of global warming on embryonic little skate, *Leucoraja erinacea* (Mitchill). J. Exp. Mar. Biol. Ecol. **463**, 72–78 (2015).

[58] D. Benetti, R. Brill, S. Kraul Jr., The standard metabolic rate of dolphin fish. J. Fish Biol. **46**, 987–996 (1995).

[59] J. B. Leonard, A. P. Summers, T. J. Koob, Metabolic rate of embryonic little skate, Raja erinacea (*chondrichthyes: Batoidea*): The cost of active pumping. J. Exp. Zool. **283**, 13–18 (1999).

[60] A. Brafield, D. Solomon, Oxy-calorific coefficients for animals respiring nitrogenous substrates. Comp. Biochem. Physiol. Part A Physiol. **43**, 837–841 (1972).

[61] T. L. Hedrick, Software techniques for two-and three-dimensional kinematic measurements of biological and biomimetic systems. Bioinspir. Biomim. **3**, 034001 (2008). 18591738 10.1088/1748-3182/3/3/034001

[62] G. Xiong, G. V. Lauder, Center of mass motion in swimming fish: Effects of speed and locomotor mode during undulatory propulsion. Zoology **117**, 269–281 (2014). 24925455 10.1016/j.zool.2014.03.002

[63] M. A. Kolmann , DiceCT for fishes: Recommendations for pairing iodine contrast agents with *μ*CT to visualize soft tissues in fishes. J. Fish Biol. **102**, 893–903 (2023). 36647819 10.1111/jfb.15320

[64] M. Fath, S. Nguyen, J. Donahue, S. McMenamin, E. Tytell, Static stability and swim bladder volume in the bluegill sunfish (*Lepomis macrochirus*). Integr. Organismal Biol. **5**, obad005 (2023). 10.1093/iob/obad005 PMC10002887 36910303

[65] A. Fedorov , 3D Slicer as an image computing platform for the Quantitative Imaging Network. Magn. Reson. Imaging **30**, 1323–1341 (2012). 22770690 10.1016/j.mri.2012.05.001 PMC3466397

[66] R Core Team, R: A Language and Environment for Statistical Computing (R Foundation for Statistical Computing, Vienna, Austria, 2021).

[67] E. Abouheif, A method for testing the assumption of phylogenetic independence in comparative data. Evol. Ecol. Res. **1**, 895–909 (1999).

[68] S. Pavoine, S. Ollier, D. Pontier, D. Chessel, Testing for phylogenetic signal in phenotypic traits: New matrices of phylogenetic proximities. Theor. Popul. Biol. **73**, 79–91 (2008). 18022657 10.1016/j.tpb.2007.10.001

[69] T. Jombart, S. Dray, adephylo: Exploratory analyses for the phylogenetic comparative method. Bioinformatics **26**, 1907–1909 (2010). 20525823 10.1093/bioinformatics/btq292

[70] F. Keck, F. Rimet, A. Bouchez, A. Franc, phylosignal: An R package to measure, test, and explore the phylogenetic signal. Ecol. Evol. **6**, 2774–2780 (2016). 27066252 10.1002/ece3.2051 PMC4799788

[71] V. Di Santo , Dataset for: Inherent instability leads to high costs of hovering in near-neutrally buoyant fishes. Dryad. 10.5061/dryad.9cnp5hqw7. Deposited 28 May 2025. PMC12280926 40623189

