# Supplementary material for: Inherent instability leads to high costs of hovering in near-neutrally buoyant fishes
Source: Proc Natl Acad Sci U S A. 2025 Jul 7;122(28):e2420015122. doi: 10.1073/pnas.2420015122 (PMC12280926; doi:10.1073/pnas.2420015122)
Supplement: Supplementary file 1 — Appendix 01 (PDF) [file pnas.2420015122.sapp.pdf]

Supporting Information for

Inherent instability leads to high costs of hovering in near-neutrally buoyant fishes

Valentina Di Santo, Xuewei Qi, Fidji Berio, Angela Albi, Otar Akanyeti

Corresponding Author: Valentina Di Santo.
E-mail: vdisanto@ucsd.edu

This PDF file includes:

Supporting text
Fig. S1
Tables S1 to S5

Supporting Information Text

Extended Results

Energetics. To quantify the energetic costs of hovering, we conducted two sets of experiments: a 30-min hover swimming ($MO_{2\ hover}$) followed by a 30-min rest ($MO_{2\ rest}$). The two separate measurements were necessary to determine the net costs of hovering, $MO_{2net} = MO_{2hover}$ - MO_{2rest}, and the ratio MO_{2hover} / MO_{2rest}), as well as compare fishes maintained at different salinities (marine, brackish, and freshwater) and temperatures (range: 18-28°C). MO_2 differed significantly across species (MO_{2hover}: $F_{(12,76)} = 9.24$, $p < 0.0001$; MO_{2rest}: $F_{(12,76)} = 5.08$, $p < 0.0001$; MO_{2net}: $F_{(12,76)} = 10.2$, $p < 0.0001$; MO_{2hover} / MO_{2rest}: $F_{(12,76)} = 6.27$, $p < 0.0001$). Across the species tested, MO_{2hover} were significantly higher than MO_{2rest} ($F_{(1,176)} = 59.03$, $p < 0.0001$). In fact, MO_2 doubled from an average of 166.97 during resting to an average of 351.37 mgO$_2$ kg^{-1} h^{-1} during hovering. Within each species, MO_{2hover} were significantly higher than MO_{2rest} (all $p < 0.05$) except for sand smelt ($MO_{2hover} = 263.40$ mgO$_2$ kg^{-1} h^{-1} vs. $MO_{2rest} = 187.72$ mgO$_2$ kg^{-1} h^{-1}, $F_{(1,10)} = 3.22$, $p = 0.1$) and three-striped gourami ($MO_{2hover} = 158.48$ mgO$_2$ kg^{-1} h^{-1} vs. $MO_{2rest} = 83.39$ mgO$_2$ kg^{-1} h^{-1}, $F_{(1,10)} = 4.14$, $p = 0.06$). Hovering costs (KJ kg^{-1}) were obtained from $MO_{2\ net}$ values and averaged per species. Hovering costs range from as low as 0.12 KJ kg^{-1} for goldfish and pufferfish, to as high as 0.94 KJ kg^{-1} for rummy-nose tetra and harlequin rasbora for ten min of hovering.

We calculated the average MO_{2net} and MO_{2hover} / MO_{2rest} and identified two groups of fishes based on the amount of energy spent compared to mean MO_2 for all the species. We identified fishes with low costs of hovering (G1: sand smelt *Atherina presbyter*, goldfish *Carassius auratus*, green Chromis *Chromis viridis*, figure eight puffer *Dichotomyctere ocellatus*, three-spined stickleback *Gasterosteus aculeatus*, sailfin molly *Poecilia latipinna*, three-stripe gourami *Trichopsis schalleri*) and high costs of hovering (G2: wrestling halfbeak *Dermogenys pusilla*, giant danio *Devario aequipinnatus*, rummy-nose tetra *Hemigrammus rhodostomus*, glass catfish *Kryptopterus vitreolus*, frog-faced cichlid *Lamprologus ocellatus*, harlequin rasbora *Trigonostigma heteromorpha*). If one of the two MO_2 fell above the average MO_2, then we placed that species in the high MO_2 group. Species in the high MO_{2net} group (wrestling halfbeak, rummy-nose tetra, glass catfish, harlequin rasbora, and giant danio) showed significantly higher MO_2 values compared to the low MO_{2net} group ($t = 9.068$, $p < 0.001$). Similarly, species in the high MO_{2hover} / MO_{2rest} group (frog-face cichlid, harlequin rasbora, and giant danio) exhibited significantly higher values compared to the low MO_{2hover} / MO_{2rest} group ($t = 4.056$, $p < 0.001$).

Kinematics. Fishes trim during hovering, resulting in complex 3D trajectories of their fin movement. Because of this, typical kinematic features such as frequency and amplitude do not accurately characterize the movement of the fins. Instead, we measured the distance traveled by the fin in a second (as a proportion of body length, BL) to estimate fin effort. We also measured whether the pectoral fins were synchronized, as often seen during steady swimming. We observed that the pectoral fin movement is synchronized, either in-phase or anti-phase across species. We observed that often the same fish switched from in-phase to anti-phase and *vice versa*. The distance traveled (BL) by dorsal, anal, and caudal fins in a second differed statistically across species (dorsal: $F_{(10,13)} = 4.89$, $p = 0.005$; anal: $F_{(12,23)} = 4.84$, $p = 0.0006$, caudal: $F_{(12,23)} = 9.48$, $p < 0.0001$), while it did not differ for pectoral ($F_{(12,23)} = 1.82$, $p = 0.1$) and pelvic fins ($F_{(7,9)} = 1.29$, $p = 0.3$). Giant danio had the highest mean fin travel distance across fins (pectoral: 7.66 BL vs. 1.90 BL for the rummy-nose tetra; dorsal: 5.15 BL vs. 0.40 BL for the molly; anal: 3.08 BL vs. 0.43 BL for the molly; pelvic: 1.54 BL vs. 0.22 BL for the gourami; caudal: 7.27 BL vs. 0.47 BL for the pufferfish). When we analyzed the distance traveled by the fins in the two MO_2 groups, we found that only the distance traveled by the caudal fin was significantly higher in the G2 (G2 mean: 3.83 BL, G1 mean: 1.76 BL; $F_{(1,34)} = 13.21$, $p = 0.0009$).

Analysis of posture during hovering showed that all fishes were dynamically stable, even though they were inherently unstable because of their morphology. Roll angle ranged from 1.71° in green chromis to 6.84° in pufferfish. Roll angle was not significantly different across species ($F_{(12)} = 0.77$, $p = 0.6$). Similarly, pitch angles were relatively small, ranging from 0.27° in gourami to 4.78° in cichlid, and were not significantly different across species ($F_{(12)} = 1.05$, $p = 0.4$). Body angle during hovering differed significantly across species ($F_{(12)} = 5.73$, $p < 0.0001$). Most fishes assumed a positive body angle (e.g., giant danio, $\theta = 23.2°$) or negative (e.g., pufferfish, $\theta = -24.4°$) relative to the horizontal plane. Some fishes maintained a nearly horizontal body posture, such as the green chromis, for example, $\theta = 0.4°$. Body curvature along the anterio-posterior axis also differed across species ($F_{(12)} = 3.41$, $p = 0.004$), and ranged from 0.008 BL in sticklebacks to 0.29 BL in molly.

Morphology. Linear and volumetric measurements were taken for each species. Mass was significantly different across species ($F_{(12)} = 45.65$, $p < 0.0001$) and ranged from 0.24 g in wrestling halfbeak to 3.2 g in pufferfish. Total length (TL) was different across species ($F_{(12)} = 16.17$, $p < 0.0001$) and ranged from 2.95 cm in the harlequin rasbora to 6.61 cm in sand smelt. Maximum body depth (MBD) was significantly different across species ($F_{(12)} = 31.53$, $p < 0.0001$) and ranged from 0.41 cm in wrestling halfbeak to 1.47 cm in giant danio. Maximum body width (MBW) was significantly different across species ($F_{(12)} = 25.83$, $p < 0.0001$) and ranged from 0.09 cm in wrestling halfbeak to 0.92 cm in pufferfish. Fineness ratios were significantly different across species ($p < 0.0001$). TL/MBD ranged from 3.03 in green chromis to 10.69 in wrestling halfbeak, while TL/MBW ranged from 4.62 in pufferfish to 46.31 in wrestling halfbeak. The separation of the COM and the COB was calculated for each species from one specimen (Supplemetary Table S1).

Model. The results of the multi-linear regression analysis are presented below. The regression equation is:

$$Y = C_0 + C_1 \times X_1 + C_2 \times X_2 + C_3 \times X_3 + C_4 \times X_4 + C_5 \times X_5 + C_6 \times X_6 + C_7 \times X_7,$$

where: Y = $MO2_{net}$ or $MO_{2\ \mathrm{hover}}$ / $MO_{2\ \mathrm{rest}}$, C_i = Coefficient for the i-th variable, and X_i = Independent Variable.

The multi-linear regression analysis shows the key factors that modulate the increase in $MO2_{net}$ and $MO_{2\ \mathrm{hover}}$ / $MO_{2\ \mathrm{rest}}$ in near-neutrally buoyant fishes. The intercept values, representing the baseline metabolic rates when all independent variables are zero, are 1501.05 for $MO2_{net}$ and 9.35 for $MO_{2\ \mathrm{hover}}$ / $MO_{2\ \mathrm{rest}}$. Notably, mass exhibits a significant negative correlation with both $MO2_{net}$ (-440.24) and $MO_{2\ \mathrm{hover}}$ / $MO_{2\ \mathrm{rest}}$ (-2.91), indicating that fishes with a larger mass exhibit lower metabolic rates. The fineness ratio of total length to maximum body depth (TL/MBD) positively correlates with both $MO2_{net}$ (201.28) and $MO_{2\ \mathrm{hover}}$ / $MO_{2\ \mathrm{rest}}$ (0.63), suggesting that a higher TL/MBD corresponds to higher metabolic costs. The fineness ratio of total length to maximum body width (TL/MBW) correlates with both $MO2_{net}$ (194.16) and $MO_{2\ \mathrm{hover}}$ / $MO_{2\ \mathrm{rest}}$ (0.21), suggesting that, for a given body length, an increase in fish width is associated with lower metabolic rates.

The distance between the center of mass and buoyancy along the anterior-posterior axis (COM-COB a-p) shows a negative correlation with $MO2_{net}$ (-114.18) and $MO_{2\ \mathrm{hover}}$ / $MO_{2\ \mathrm{rest}}$ (-0.34). Similarly, the distance along the dorsal-ventral axis (COM-COB d-v) also shows negative correlations with $MO2_{net}$ (-116.80) and $MO_{2\ \mathrm{hover}}$ / $MO_{2\ \mathrm{rest}}$ (-0.80). We note that the COM-COB in many fishes is negative as the COB is often below (d-v) or posterior (a-p) to the COM, so a negative correlation suggests that a larger COM-COB distance increases $MO2_{hover}$. The position of the pectoral fin shows a negative correlation with both $MO2_{net}$ (-1297.36) and $MO_{2\ \mathrm{hover}}$ / $MO_{2\ \mathrm{rest}}$ (-3.94). Similarly, the caudal fin position shows a negative correlation with both $MO2_{net}$ (-1297.60) and $MO_{2\ \mathrm{hover}}$ / $MO_{2\ \mathrm{rest}}$ (-6.97).

The model accounts for 86% and 63% of the variability in $MO2_{net}$ and $MO_{2\ \mathrm{hover}}$ / $MO_{2\ \mathrm{rest}}$, respectively. These findings show that fin positions, mass, and shape (fineness ratios) are primary determinants of $MO2_{net}$ and $MO_{2\ \mathrm{hover}}$ / $MO_{2\ \mathrm{rest}}$, offering insights into the energetic and biomechanical constraints of hovering in fishes that are small, have a large COM-COB, and that have fins closer to the anterior of the body, as these are more likely to incur higher metabolic costs.

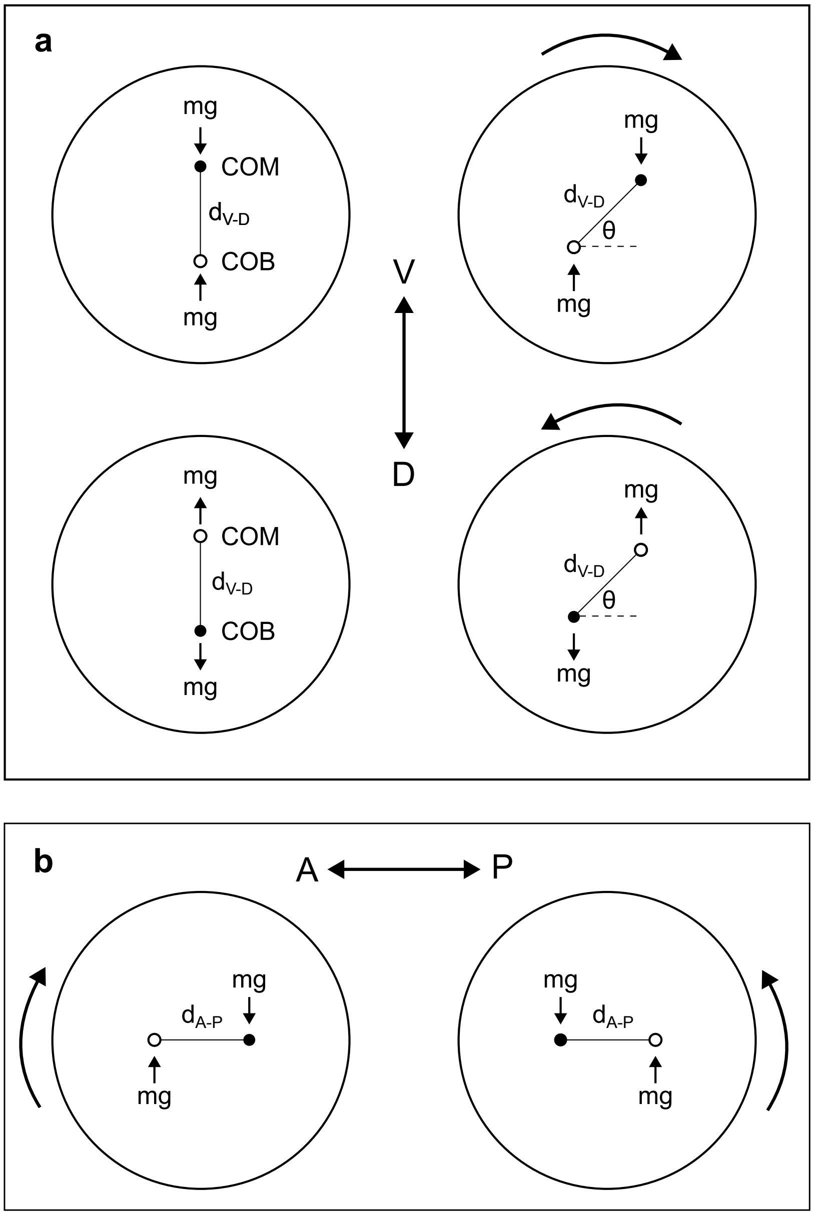

Fig. S1. A schematic diagram illustrating intrinsic instability of a disk-shaped body caused by the separation between COM and COB. **a. Separation between COM and COB along the ventral (V) – dorsal (D) axis (d_{V-D}).** If COB is lower than COM (i.e., $d_{V-D} < 0$), a small perturbation (i.e., angular displacement, θ) around the anterior (A) – posterior (P) axis would create a torque, $\tau_{\text{COM-COB}(V-D)}$, proportional to $m \times g \times d \times \cos(\theta)$, and make the body roll upside down (m: mass and g: gravitational force). Hence, corrective actions are required to remain upright (i.e., unstable condition). On the other hand, if COB is higher than COM (i.e., $d_{V-D} > 0$), the same perturbation would be compensated without requiring corrective actions (i.e., stable condition). **b. Separation between COM and COB along the anterior (A) – posterior (P) axis.** This would create a torque, $\tau_{\text{COM-COB}(A-P)}$, around the mediolateral axis (left-right axis). This time, $\tau_{\text{COM-COB}(A-P)}$ will be proportional to pitch angle instead of roll angle. If COB is positioned more anteriorly than COM, the body would pitch up (left), and if COB is positioned more posteriorly (right), the body would pitch down until it is vertical in the water column. In both cases, $\tau_{\text{COM-COB}(A-P)}$ is proportional to $|d_{A-P}|$, and corrective actions are required to maintain stable pitch (i.e., unstable condition).

Table S1. Morphological characteristics of species and energy spent to hover for 10 minutes.

Species	Mass (g)	TL (cm)	TL/MBW	TL/MBD	COM-COB (a-p)	COM-COB (d-v)	$\text{Energy}_{\text{hover}}$ (KJ)
Atherina presbyter (Sand Smelt)	1.92	6.61	10.78	7.55	-0.02	0.01	0.17
Carassius auratus (Goldfish)	3.14	5.88	10.91	3.87	-0.14	0.04	0.13
Chromis viridis (Green Chromis)	0.87	4.12	8.75	3.04	0.04	-0.13	0.22
Dermogenys pusilla (Wrestling Halfbeak)	0.24	4.27	46.31	10.70	-0.08	0.08	0.78
Devario aequipinnatus (Giant Danio)	1.34	6.13	13.44	3.95	-0.02	-0.07	0.42
Dichotomyctere ocellatus (Figure Eight Puffer)	3.20	4.29	4.62	3.31	-0.11	-0.37	0.13
Gasterosteus aculeatus (Three-spined Stickleback)	1.04	4.63	9.33	5.23	-0.21	0.06	0.21
Hemigrammus rhodostomus (Rummy-nose Tetra)	0.34	3.15	9.70	4.46	-0.06	-0.16	0.95
Kryptopterus vitreolus (Glass Catfish)	0.93	4.90	12.22	5.46	0.16	-0.21	0.72
Lamprologus ocellatus (Frog-faced Cichlid)	1.13	4.35	7.04	3.90	-0.02	-0.30	0.39
Poecilia latipinna (Sailfin Molly)	0.89	3.97	6.94	4.22	-0.15	0.15	0.19
Trichopsis schalleri (Three-stripe Gourami)	4.90	3.76	10.98	4.23	0.18	0.07	0.17
Trigonostigma heteromorpha (Harlequin Rasbora)	0.57	2.96	9.74	3.50	0.00	0.07	0.94

Mass (g; n = 4–8), TL = Total Length (cm; n = 3), TL/MBW = Total Length to Maximum Body Width ratio (n = 3), TL/MBD = Total Length to Maximum Body Depth ratio (n = 3), COM-COB (a-p) = distance between the center of mass and buoyancy on the anterior-posterior axis as a proportion of body length (n = 1), COM-COB (d-v) = distance between the center of mass and buoyancy on the dorsal-ventral axis as a proportion of body depth (n = 1), $\text{Energy}_{\text{hover}}$ (KJ) for 10 min (n = 4–8). All values are means.

Table S2. Results of Abouheif's tests performed to investigate the existence of a phylogenetic signal among six energetic, morphological, and kinematics measurements. The results show that pectoral fin position along the anterior-posterior axis of fishes is phylogenetically structured.

Variable	C-statistic	p
$MO_{2\ hover}$ / $MO_{2\ rest}$	0.022	0.288
$MO_{2\ net}$	0.124	0.154
COM - COM A-P Axis	-0.052	0.376
COM - COB D-V axis	-0.129	0.557
Pectoral fin A-P axis	0.470	**0.005***
Caudal fin A-P axis	0.022	0.246

A-P, anterior-posterior; COB, center of buoyancy; COM, center of mass; D-V, dorsal-ventral. The tests are performed on means.

Table S3. Correlation coefficient among input variables: x_1 - Mass; x_2 - Fineness ratio (TL/MBD); x_3 - COM-COB (A-P); x_4 - COM-COB (D-V); x_5 - Fineness ratio (TL/MBW); x_6 - Pectoral fin position; x_7 - Caudal fin position.

	x_1	x_2	x_3	x_4	x_5	x_6	x_7
x_1	1	-0.25	-0.34	-0.32	-0.35	0.22	-0.33
x_2		1	-0.13	0.41	**0.76***	0.18	0.46
x_3			1	-0.21	-0.03	-0.44	-0.11
x_4				1	0.33	-0.16	-0.24
x_5					1	0.19	0.30
x_6						1	0.30
x_7							1

*p < 0.01, other correlations are not statistically significant.

The strongest correlation is observed between x_2 (Fineness ratio TL/MBD) and x_5 (Fineness ratio TL/MBW) ($R^2 = 0.76$, $p < 0.01$), indicating a strong association between these two morphological descriptors. Other correlations are not statistically significant, suggesting limited interdependence among the remaining variables.

Table S4. Variable importance in predicting MO$_2 net$ and MO$_2 hover$ / MO$_2 rest$, measured by the resulting change in model performance (ΔR^2). Larger reductions indicate greater variable importance. The "Remove" condition assesses the effect of excluding a variable entirely, while the "Shuffle" condition evaluates the impact of randomizing a variable's values, disrupting its predictive contribution. Mass (x_1), COM-COB (D-V) (x_4), and pectoral fin position (x_6) emerged as the most influential morphological predictors of metabolic rates.

	R^2 (MO$_2$ net)	R^2 (MO$_2 hover$ / MO$_2 rest$)	ΔR^2 (MO$_2$ net)	ΔR^2 (MO$_2 hover$ / MO$_2 rest$)
Original	0.86	0.63		
**Remove**				
x_1	0.56	0.39	-0.30	-0.24
x_2	0.84	0.63	-0.02	0
x_3	0.72	0.61	-0.14	-0.02
x_4	0.64	0.44	-0.22	-0.19
x_5	0.83	0.63	-0.03	0
x_6	0.67	0.60	-0.19	-0.03
x_7	0.81	0.61	-0.05	-0.02
x_2, x_5	0.67	0.62	-0.19	-0.01
**Shuffle**				
x_1	0.60	0.48	-0.26	-0.15
x_2	0.85	0.64	-0.01	0.01
x_3	0.74	0.63	-0.12	0
x_4	0.70	0.57	-0.16	-0.06
x_5	0.84	0.63	-0.02	0
x_6	0.77	0.61	-0.09	-0.02
x_7	0.84	0.61	-0.02	-0.02
x_2, x_5	0.71	0.63	-0.15	0

Table S5. Multi-linear regression coefficients for MO$_2net$ and MO$_2hover$ / MO$_2rest$ (reduced input set).

Coefficient Variable (X)	MO$_2net$ (Y)	MO$_2hover$ / MO$_2rest$ (Y)
C_0 = Intercept	531.69	4.0
C_1 = Mass	-307.43	-2.21
C_2 = COM – COB (A-P)	-81.21	-0.16
C_3 = COM – COB (D-V)	-76.94	-0.61
C_4 = Fineness ratio (TL/MBW)	270.57	0.35
C_5 = Pectoral fin position	-1438.78	-4.78
R^2	0.8	0.59
